# Nitrous Oxide as an Anæsthetic

**Published:** 1866-01

**Authors:** 


### NITROUS OXIDE AS AN ANÆSTHETIC.

The use of nitrous oxide gas as an anæsthetic in surgery appears to be rapidly increasing in favor. In a number of capital operations, recently performed in this city, it has been satisfactorily used, and the anæsthesia being as complete as that from ether or chloroform.

At one of the clinics of the Ophthalmic Hospital, Dr. Levis remarked that, in the present state of information in regard to its anæsthetic effects, and with the inefficient means of administration, he thought its applicability to surgery very valuable, but limited. It is yet to be determined whether insensibility from nitrous oxide can safely be prolonged during operations which require much time for their performance. The continuance, too, of anæsthesia while inhalation is effected with the usual tubular mouth-piece, requires the voluntary effort of the patient, which may not always be attainable, especially with children and with persons making resistance under temporary excitement. He believed that it might well displace ether and chloroform in most operations of short duration, and in this class could be included the amputations. A decided advantage, particularly for administration at the clinics, is the rapidity with which anæsthesia is induced, and also the speedy revival of the patient without depression or nausea.

In many operations in ophthalmic practice it is applicable, but for the operation of extraction of cataract its anæsthesia has not the relaxed and tranquil character which is desirable.

The preparation of the gas by decomposition of the nitrate of ammonia by heat, and its subsequent purification, are simple, and the cost is probably not one-fourth that of other anæsthetics.

The present intelligent attention to the subject will soon give a proper appreciation and fix the status of nitrous oxide as an anæsthetic, but we can already, from the present evidence, commend it to practitioners for its safety, agreeableness to patients, rapidity of effects, and economy of use.

For a knowledge of this remarkable agent in all its bearings, we refer those who are interested, to the exhaustive monograph on "Nitrous Oxide," by Dr. ZEIGLER, recently published in this city.

